# Characterizing the semantic and form-based similarity spaces of the mental lexicon by means of the multi-arrangement method

**DOI:** 10.3389/fpsyg.2022.945094

**Published:** 2022-08-11

**Authors:** Lukas Ansteeg, Frank Leoné, Ton Dijkstra

**Affiliations:** Donders Institute for Brain, Cognition and Behaviour, Radboud University Nijmegen, Nijmegen, Netherlands

**Keywords:** similarity, representational similarity analysis, multi-arrangement, multimodal, semantics, phonology, orthography, mental lexicon

## Abstract

Collecting human similarity judgments is instrumental to measuring and modeling neurocognitive representations (e.g., through representational similarity analysis) and has been made more efficient by the multi-arrangement task. While this task has been tested for collecting semantic similarity judgments, it is unclear whether it also lends itself to phonological and orthographic similarity judgments of words. We have extended the task to include these lexical modalities and compared the results between modalities and against computational models. We find that similarity judgments can be collected for all three modalities, although word forms were considered more difficult to sort and resulted in less consistent inter- and intra-rater agreement than semantics. For all three modalities we can construct stable group-level representational similarity matrices. However, these do not capture significant idiosyncratic similarity information unique to each participant. We discuss the potential underlying causes for differences between modalities and their effect on the application of the multi-arrangement task.

## Introduction

When language users process or learn a target word, other lexical representations that are similar are temporarily activated in the mental lexicon, competing with the target for recognition (Morton, 1969; Marslen-Wilson and Welsh, 1978; Davis, 2010). For instance, a Dutch auditory input like/kapi/leads to the coactivation of the phonological and semantic representations of both/kapitein/(“captain”) and/kapitaal/(“capital”) (Zwitserlood, 1989). As a consequence, the similarity structure of the mental lexicon in terms of form and meaning plays an important role in understanding language usage and word learning. Such lexical similarity is multidimensional, as it can be assessed with respect to phonological, orthographic, and semantic dimensions (e.g., Seidenberg and McClelland, 1989). This raises the question whether similarity is similarly processed in each modality and can be measured in the same way for each.

In this study, we investigate the role of similarity in each of the three modalities of vocabulary, examining aspects that overlap or are disjoint between modalities. Can all or any modalities be considered as similar, well-defined metric spaces, or are they, in fact, structurally different? For our analysis, we adapt the multi-arrangement method (Kriegeskorte and Mur, 2012) and apply it as a multimodal approach for collecting human similarity judgments. This will allow us to consider the usefulness of this method for vocabulary similarity characterization.

To set the stage for our research, we first consider how earlier studies have conceived of similarity spaces in general and those of lexical form and meaning more specifically. Next, we describe the multi-arrangement method and evaluate its application to different modalities. We analyze both the shape of distributions of similarity judgments within modalities and the relationship between modalities.

To understand how representations of words and concepts are acquired, the theoretical notion of psychological space has been coined. In one of its first formal descriptions, Shepard (1987) proposed that stimuli are represented in a metric space as points or regions, while the spatial dimensions represent perceptual features. Perceptual input stimuli are generalized to the region or the closest concept (i.e., the coordinates of their perceptual features in psychological space). Confusion may arise in overlapping regions or when multiple concepts are positioned close together in space. When perceiving a furry animal with four legs in a residential neighborhood, it is easy to confuse a fox for a cat until a feature is recognized that places the animal in a region of this space uniquely assigned to the concept “fox.” “Closeness” in such spaces appears to follow an exponential function: Items that are very close in this space are perceived as similar, while slightly less close items are already perceived to be dissimilar. Although foxes and raspberries share some features (both are alive, red, and usually found in forests) a human rater will likely classify them as entirely dissimilar.

Analyzing the shape and structure of psychological spaces is difficult, because in experiments we can at best interact with them in indirect ways. The report of a participant on the perceived similarity of two items is subject to task strategies, context aspects, and noise. Additionally, reported similarity may differ across participants, because it concerns both a “common space” and unique individual tendencies (Carroll and Chang, 1970). Thus, although an underlying metric psychological space can be modeled, human-reported similarities might, according to Shepard, reflect a warped perception of those representational distances. Different methods to assess this space, such as pairwise human judgments, priming effects, or neuroimaging measures may be subject to different biases toward the psychological space. This may produce different results when two different tasks are designed to assess the same underlying space characteristics.

Similarity plays an important role not only with respect to representational aspects of language, but also its processing. It has long been known that competitor sets of similar words are initially activated when people are learning and using a particular target word (Andrews, 1989; Grainger, 1990). Put differently, exposure to a word results in the co-activation of similar and related words. These competitor sets are called neighborhoods in the visual modality (Grainger, 1990) and cohorts in the auditory modality (Marslen-Wilson, 1987), but the similarity set may also be semantic in nature (Hantsch et al., 2005; Zhuang et al., 2011). Coactivation and subsequent competition of items can result in facilitatory or inhibitory effects in tasks like translation production, (primed) lexical decision, and word naming (Dijkstra et al., 2010). Effects are found across both meaning and form modalities. For instance, semantically similar words can exert facilitatory effects even in tasks where meaning is not critical to task performance (Rommers et al., 2013). Items that are similar in form interact both in visual (Schwartz et al., 2007) and auditory (Marian and Spivey, 2003) tasks based on their written and spoken forms.

These effects are not only relevant for the understanding of underlying mental processes, but have direct practical implications for vocabulary acquisition. The ease of learning a new word is strongly affected by its similarity to known words both in a learner’s native language (L1) and other languages (L2, L3, and so on) (Schepens et al., 2016). Contrasting similar words may help learners to distinguish and internalize them (Baxter et al., 2021), while confusing them results in errors.

In sum, understanding similarity is important for assessing processing and learning. Being able to model similarity in detail is crucial for computational models designed for language learning applications. For example, the Multilink model for printed word retrieval by monolinguals and bilinguals (Dijkstra et al., 2019), based on the earlier BIA + (Dijkstra and van Heuven, 2002) and IA models (McClelland and Rumelhart, 1981), implements words as network nodes that are activated on the basis of similarity to the input item. Because activated items compete for selection in recognition, the implementation of a similarity metric, in this model Levenshtein distance, is crucial to the model’s predictions about word retrieval. A computational metric of similarity is also important for learning methods such as contrasting in intelligent tutoring systems (Baxter et al., 2021).

Semantic similarity measures have been widely gathered (e.g., Hill et al., 2015) and are often modeled by using one of two foundations: semantic feature dimensions, or word co-occurrence. Semantic features define concepts in terms of constellations of important characteristics (Collins and Quillian, 1969). The features that are deemed relevant are often hand-picked and labeled by researchers. Multidimensional similarity spaces based on word co-occurrence are generated through natural language processing algorithms such as Latent Semantic Analysis (Deerwester et al., 1990) or Word2Vec (Mikolov et al., 2013). These approaches are able to capture semantic dimensions that are not predefined and can also avoid manual labeling of all items for each feature. They also capture feature-based models seem to outperform co-occurrence-based models in relation to word processing behavior in human participants (Crutch et al., 2013). However, a semantic space structured by predefined features seems to work less well for abstract concepts; it might be restricted to concrete words that can be related to motor-sensory experience (Wang et al., 2018).

Relatively little research has been done on human similarity judgments of written and spoken words, although some pairwise judgments have been collected. Examples are the work by Tokowicz et al. (2002) and Dijkstra et al. (2010) for translation equivalents. The first study reports a very high agreement between orthographic and phonological similarity ratings for English (0.96). Tokowicz et al. even collapse the two rating types into a general form similarity index. In these studies, a strong divide between item pairs judged as similar vs. dissimilar has been found between cognate (translation equivalents with form overlap) and non-cognate item pairs (with the latter accounting for the majority of items). This is in line with Shepard’s observations about exponentially decaying perceived similarity and has also been reported in studies with more fine-grained levels of dissimilarity in their stimulus selection.

Whereas semantic similarity is often assessed on the basis of meaning features or co-occurrence statistics, the similarity of orthographic and phonological word forms is more often derived algorithmically in terms of overlap in graphemes and phonemes (abstract representations of letters and speech sounds, respectively). A common measure for dissimilarity between written words is the “normalized Levenshtein distance” (Levenshtein, 1966) that measures the number of additions, deletions, and/or substitutions to derive one string from another, divided by the string length (e.g., Dijkstra et al., 2019). This measure can also be applied to lexical-phonological representations (Gooskens et al., 2008). The characteristics of orthographic similarity differ across scripts, where alphabet-based languages can use letters (or graphemes) as units of comparison, while scripts such as Japanese kanji might be better modeled by counting radicals (e.g., Yencken and Baldwin, 2006).

The performance of models is usually assessed in comparison to human similarity judgments. Judgments for model evaluation can be collected with this goal in mind or taken from datasets such as SimLex-999 (Hill et al., 2015). Although human judgments are considered as a “golden standard” for evaluation, some models now outperform humans in terms of inter-rater reliability when it comes to similarity assessment (Richie et al., 2019). This outcome is in part due to imperfect task performance by human participants, as well as different ways to interpret similarity as hierarchical, categorical, related, or alike. Less than perfect inter-rater agreement might also point to personal differences in internal representations. What seems similar to one person might seem different to another based on personal experience, no matter how good the test. To illustrate, most European participants are unlikely to group jellyfish with food items, whereas some Asian participants might readily associate them.

In most studies mentioned above, human similarity judgments were collected by asking participants to rate the similarity of word pairs on a Likert scale. While this is the most straightforward procedure, it is time consuming, and the resulting datasets often consist of relatively few pairs sampled from a limited number of participants. More time efficient methods include categorizing items or sorting them on a 2D surface (Richie et al., 2020). The latter method allows judging similarities of multiple pairs at once, albeit at a loss of accuracy for each given pair.

To reduce the loss of specificity, Kriegeskorte and Mur (2012) introduced a multi-arrangement task that efficiently collects similarity judgments from participants, and the inverse multidimensional scaling (IMDS) algorithm that combines these trials into a single representational dissimilarity matrix (RDM). This method has been demonstrated to collect judgments more efficiently than pairwise or free sorting tasks, and, crucially, to be able to account for high-dimensional similarity spaces. This is because different trials may reveal different item relationships due to the specific context of the trial set. For instance, in the context of various winged or aquatic animals, chickens and salmon might be judged to be very dissimilar, whereas a set with many edible items might result in a closer proximity of salmon to chicken than to sea creatures the participant does not consider edible. Even though participants arrange each stimulus set on only two dimensions, the combination of multiple subsets allows for extrapolation to multiple dimensions through the inverse dimensional scaling method. By contrast, multiple trials of pairwise judgments would result in multiple measures of the same one-dimensional similarity.

The efficiency of the multi-arrangement task in terms of pairs judged per participant in a given time also allows the creation of individualized models, which have been used, for example, to predict neuroimaging results (Charest et al., 2014). Slower methods such as pairwise judgments would take a long time to generate a complete RDM for an individual participant. The multi-arrangement task can therefore also be considered useful in investigating the degree to which there might be a shared common space of similarity that participants universally agree upon, and to which degree individual ratings are based on idiosyncratic representations (Charest and Kriegeskorte, 2015).

The multi-arrangement task has been successfully applied to the study of general semantic similarity effects (Charest et al., 2014; Fang et al., 2018; Majewska et al., 2020). It has not been tested on the orthographic and phonological modalities of vocabulary, which might rely on different underlying psychological spaces. As the multi-arrangement task would be a useful tool to compare similarity data across the modalities of vocabulary, we examine whether it can be applied to word form similarity as well as it can to semantic information.

In this study, we will apply the multi-arrangement task to compare similarity spaces across the modalities of semantics, phonology, and orthography. By collecting similarity judgments for all three modalities on one and the same set of words, we intend to chart structural differences between the organization of their psychological spaces. This may then pave the way for finding more appropriate metrics to simulate and measure these spaces.

We will evaluate the application of the multi-arrangement task across the three dimensions to establish whether the task can be extended to word form similarities. We will confirm our operationalization of the three modalities by comparing the resulting data to existing models: Word2Vec for semantic similarity, and Levenshtein distance of letters and phonemes for orthography and phonology, respectively.

We have three hypotheses. First, we expect the multi-arrangement method to allow for consistent measurement of all three modality-specific similarity spaces, within and over participants. Second, we expect the two form-oriented conditions to be more distinct from semantics than from each other, because Dutch, as used here, has a relatively shallow script in which orthographic and phonological forms of words are inherently strongly correlated (Dijkstra et al., 2010). Third, we expect more consistent measures of group-level similarity than between subjects, as similarity measures are expected to be highly idiosyncratic for all modalities.

## Materials and methods

### Participants

Seventy-three participants completed three online sorting sessions. All participants were native speakers of Dutch recruited through the Radboud University SONA participant system and compensated with study credits or 30 euros for participation. Participants gave informed consent in accordance with guidelines of the Radboud University Social Sciences Ethics Committee (ECSW-2018-115).

The data of 18 participants were excluded from further analysis for the following reasons. Ten participants misunderstood instructions or sorted on the wrong modality in at least one session (not uncommon in online experimental context), as confirmed by visual inspection of their trials and their self-described task strategies in the post-survey. Eight participants provided fewer than six trials for at least one of the sessions, thus providing too little data to construct a reliable IMDS estimate. Therefore, in total 55 native Dutch speakers (44 female, mean age: 22.6 years) were included in the analysis.

### Stimulus materials

**FIGURE 1:**
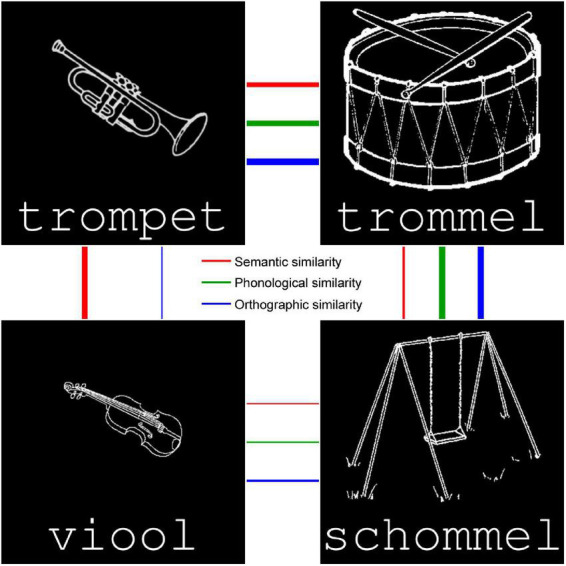
Four example stimuli, showing both the drawn image and typed word (semantics and orthography, respectively). Auditory versions of the spoken word are played when the stimulus is clicked or dragged, to activate phonology. The thickness of the arrows indicates semantic, orthographic, and phonological similarity between items (thicker arrows refer to more similar items).

Participants took part in the online experiments on laptops or PCs with a screen of at least 13” diagonally, using trackpad or mouse to interact with the experiment. In all three sessions, they sorted items that each corresponded to one word. The items showed a line drawing and written word, and played the spoken word when clicked or dragged (Figure 1). The stimulus always consisted of all three modalities (image, written word, and spoken word) regardless of the target modality of the session, as participants were unable to sort purely auditory stimuli during the pilot. To avoid disparity between modalities by adding a visual clue to the auditory condition but no auditory clue to the visual conditions, we instead kept the stimuli invariant and only distinguished modality via task instructions. As stimuli, 70 Dutch nouns, including their picture, were selected from the international picture naming project (Székely et al., 2003). Auditory versions of the stimulus names were recorded by a female Dutch native speaker.

### Stimulus selection

The nouns were selected on the basis of imageability and lack of polysemy in Dutch, and provided a varied spectrum of semantic, orthographic, and phonological sets. The set as a whole was selected to maximize differences in similarity across modalities, i.e., it included semantically similar but orthographically dissimilar words (and vice versa), semantically similar but phonologically dissimilar words (and vice versa), and, as far as possible in Dutch, orthographically similar but phonologically dissimilar words (and vice versa). For example, in Figure 1, *trompet* and *trommel* are similar in meaning and form, *trompet* and *viool* are similar in meaning but not form, *trommel* and *schommel* are similar in form but not meaning, and *viool* and *schommel* are dissimilar in both meaning and form. Although orthography and phonology are closely linked in the shallow Dutch script, *trompet* and *trommel* are somewhat closer than *trOmpEt* and *trOm@l*, whereas *trOm@l* and *sxOm@l* are closer than *trommel* and *schommel* in terms of Levenshtein distance. This selection was guided by the computational models described in section “Computational models.”

**FIGURE 2:**
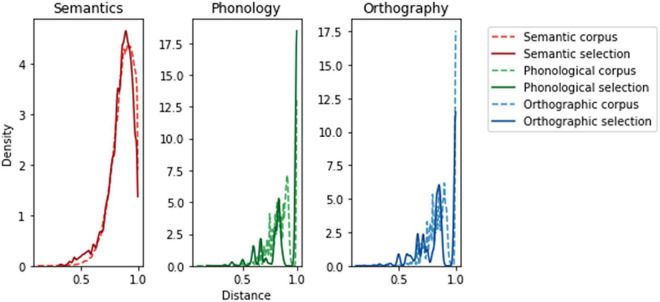
Comparison of the distributions of distances in our sample of items within the larger corpus to our stimulus selection, showing high congruence. The corpus distributions were calculated using 78.120 pairs (between 280 unique words) from our sources for semantic, phonological and orthographic model representations (see under “Computational models”). The y axis scale differs between modality as the integral of a probability density function equals 1, and the “peaks” in the form modalities are narrower due to a discrete amount of possible Levenshtein values for the word lengths in our set. The shape of the distribution is more informative than the absolute y values. The word selection favors slightly more close pairs than the corpus as seen by the larger area under the left part of each modality’s selection curve.

To confirm the representative nature of the word selection, we checked the overall distribution of similarities between item pairs according to models (see model section below). This turns out to be comparable to that of the overall lexicon, while the selection includes slightly more similar pairs than a random sample would (see Figure 2). More information on word selection and stimulus creation can be found in Supplementary Materials.

### Procedure

Participants took part in three online sessions in randomized and counterbalanced order, one for each of the three modalities: semantics, phonology, and orthography. In each session participants completed as many trials as possible in 50 min. Each session began and ended with a short questionnaire. In this paper, “session,” “condition,” and “modality” are used depending on context, but can be treated as interchangeable.

**FIGURE 3:**
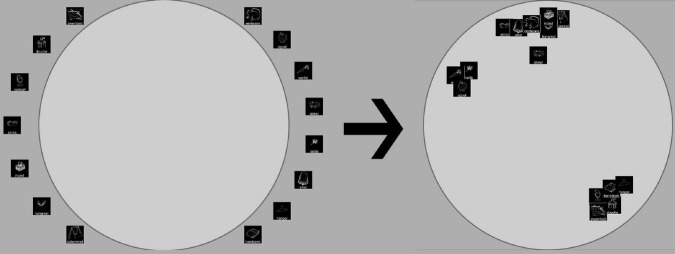
The experimental setup: Participants are presented with a set of stimuli around a circle as seen on the left and then sort these items by similarity to create an arrangement as seen on the right.

Participants performed an online implementation of the multi-arrangement task (Kriegeskorte and Mur, 2012). In this task, for each trial (arrangement), a varying number of stimuli is sorted within a circle in terms of item similarity, as shown in Figure 3. The first trial asks participants to sort the entire stimulus set. Based on how much evidence has been collected for each pair of stimuli, the program selects the subset that is most likely to provide the most additional evidence for each subsequent trial. For a detailed breakdown of the method, see Kriegeskorte and Mur (2012).

After the initial trial, the method “zooms in” on increasingly smaller sets to collect more information about subsets of the stimuli. The algorithm considers evidence collected for each pair of stimuli to be a function of distance on screen: Items that are close together are considered noisy, whereas large distances are considered as more evidence from a single trial. Although this algorithm possesses no prior information about the nature of the stimuli, a trial subset often roughly represents a category of items, such as all animals or all words ending in “er.” This is a consequence from the participant sorting these items in a cluster on an earlier trial: The algorithm picks up on this cluster and now tries to collect more information about the relative similarities of its constituent items.

The multi-arrangement task differs between the three sessions only in its instructions. The stimuli are always the same combination of image, written word, and spoken word. Participants are instructed to focus on only one modality in each session and to sort based only on that modality. We chose not to present single-modality stimuli for each condition, because participants were unable to effectively sort auditory stimuli without visual clues for each item in pilot trials. We hence presented the stimuli consistently multi-modally throughout all conditions of the experiment. After each session, the participants were asked to rate how hard they found the task on a 5-point scale, with 5 being most difficult.

### Preprocessing of the representational dissimilarity matrices

For each participant and modality, we calculated a Representational Dissimilarity Matrix (RDM) by combining the data from all trials using the IMDS algorithm. For a detailed description of this process, see Kriegeskorte and Mur (2012) or the code repository (see data availability statement). At the end of this process, we obtained a number of stimuli * number of stimuli (70*70) RDM, where each value between 0 and 1 corresponds to the dissimilarity between the column item and row item. The matrix is mirrored over its diagonal and the diagonal values are 0, as each item has no distance to itself.

**FIGURE 4:**
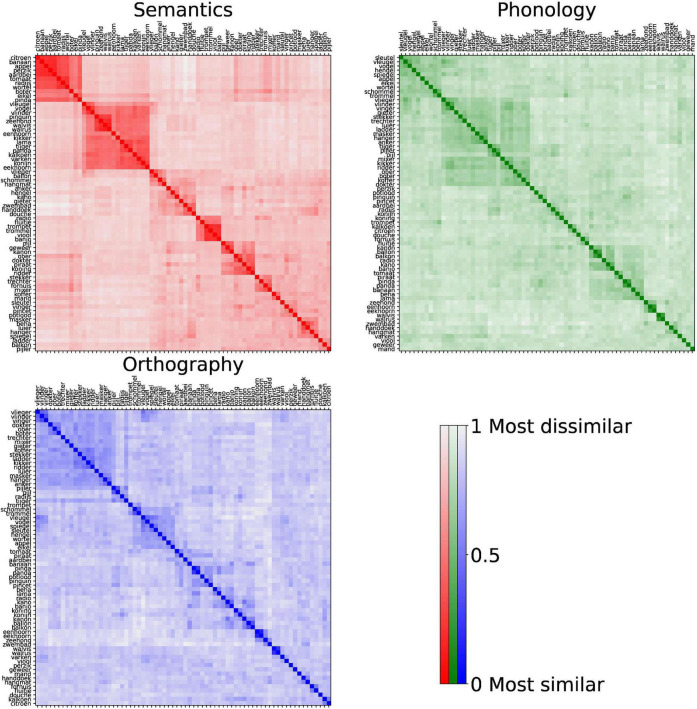
The group-level RDMs for each modality. Color indicates the degree of similarity (more saturated means more similar). The rows and columns have the same order and are sorted by hierarchical clustering, causing similar items to be adjacent, revealing the similarity clusters (saturated squares in the figure).

We also calculated a group average for each modality (see Figure 4 in results), taking the mean dissimilarity for each word pair. In analyses where individual participants were compared to the group average, the data of the participant in question were excluded from the average.

### Computational models

To validate whether we successfully operationalize the modalities, we compared our results to existing models. We used precomputed distances for English translation equivalents from a Word2Vec model of the Google News dataset (Google News Corpus, 2013) as comparison for semantic dissimilarity. For phonology and orthography, we compared measured dissimilarities to normalized Levenshtein distance on phonemes and letters, with phonemes of the PhonCLX format collected from the CELEX database (Max Planck Institute for Psycholinguistics, 2001). The model dissimilarities can also be found on the code repository.

Although we use these models to evaluate the data collected in this study, they do not represent the “ground truth.” We primarily use the models to test our operationalization, that is, to confirm that participants indeed sort on the modality we want them to. Particularly in the case of semantics, there is ongoing debate on how co-occurrence based models relate to human cognition (Günther et al., 2019). For a more robust test of our data, we also compared them against alternative models; the results of these comparisons can be found in the Supplementary Materials. This includes a Word2Vec model trained on Dutch corpus, semantic feature norms, image classification, bigram models, and spatial coding models, all of which were run as *post hoc* analyses.

## Results

To assess the application of the multi-arrangement, using the IMDS algorithm, task to semantic, orthographic, and phonological item similarity, we will now characterize the collected data patterns, subject them to analyses evaluating the consistency of similarity spaces, and, finally, compare the obtained similarity structures for different modalities.

### Task performance within and across modalities

Because participant performance was limited by time, rather than by a fixed number of trials, we first determine the number of trials that participants completed across modalities. Participants created on average 20.8 [SD = 8.6] trials in the semantic condition, 16.5 [SD = 8.4] in the phonological condition, and 14.7 [SD = 7.7] in the orthographic condition. The higher number of completed trials in the semantic condition is reflected in the reported difficulty of the task after each session: 2.84 [SD = 0.93] for semantics, 3.40 [SD = 0.98] for phonology, and 3.64 [SD = 0.92] for orthography. The semantic task was rated as significantly easier by participants than sorting on phonology [*t*(55) = -3.06, *p* = 0.0028] and orthography [*t*(55) = -4.49, *p* < 0.0001], with no significant difference between orthography and phonology.

Participants employed different strategies while completing the task, as indicated by self-reported task strategies and confirmed by visual inspection of individual arrangements. One example strategy is initially grouping items into categories and stacking items within a category on top of each other. However, a cluster analysis of individual participants found no clusters within which inter-participant agreement was significantly better than over the whole group, suggesting that task strategies did not strongly affect the resulting RDMs.

Visual inspection of the group-level RDMs (Figure 4) per modality shows that clusters (squares along the diagonal) emerged from participants’ trials. In the semantic RDM, fruits form a strong cluster with other edible items nearby, and all animals are part of a large cluster that contains sub-clusters for flying and sea-based animals. For phonology and orthography, the data are clearly less clustered. The clusters that do appear are mainly based on word ending, and position rhyming words closest to each other. Only a few word pairs, such as “eenhoorn” and “eekhoorn,” and “pinda” and “panda,” displayed similarity values comparable to semantic items within a categorical cluster.

### Consistency of similarity spaces

**FIGURE 5:**
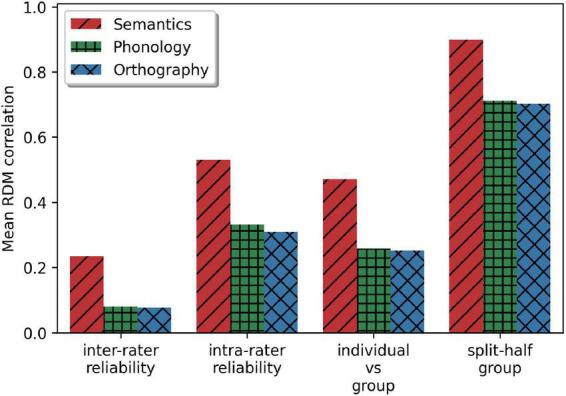
Four measures for comparison of the modalities: correlation between participants, within participants, between participants and group level, and split-half reliability of the group-level RDMs.

Next, we evaluated whether the multi-arrangement task provides consistent data for all three modalities (Figure 5), given that our method applies the task to novel similarity dimensions. Inter-rater agreement was measured by computing the Pearson correlation of the RDM estimate between any two participants, noted here with 95% confidence intervals, all significant at *p* < 0.001: *r* = 0.235 [0.232,0.238] for semantics, *r* = 0.080 [0.078,0.082] for phonology and *r* = 0.077 [0.075,0.079] for orthography. Similar results were obtained when only participants were compared who created many trials (>20) during their session. This finding suggests that the effects represent high variability between individual similarity measures, with semantic being most consistent, and are not due to an insufficient number of trials. Note that we are using Pearson correlation for this and many of the following analyses, primarily for comparability of our results to similar studies in the field. For any reported correlation value, we also included the Spearman’s rho in the Supplementary Materials. Results differ nominally, but display all the same trends as reported in this results section.

To test whether subjects were internally consistent in their responses, we calculated an alternative intra-rater agreement. Note that a regular intra-rater agreement cannot be calculated, as participants never arrange the same set of items twice, even though the same pairs of words appear in multiple trials. Instead, we calculated the predictability of a left-out trial based on all other trials of the participant for every trial other than the first trial for each participant and each modality. This is necessarily calculated by correlating the subset of the stimuli on each trial, which tends to consist of 15–25 of the 70 stimuli, rather than the full 70 stimuli used in the other measures. This calculation resulted in “intra-participant” agreement of *r* = 0.530 [0.518,0.543] for semantics, *r* = 0.332 [0.317, 0.346] for phonology and *r* = 0.309 [0.293, 0.326] for orthography. This shows, as expected, that variability is higher between than within subjects.

We compared the individual RDMs to group averages to further assess to what extent a shared similarity space is present. Each participant’s RDM estimate correlated with the group-level average RDM: semantics (*r* = 0.471 [0.448,0.495]), phonology (*r* = 0.258 [0.230,0.285]), and orthography (*r* = 0.252 [0.223,0.281]), comparable to the intra-rater reliability. In fact, the group level estimates were fairly stable, as shown by the split-half reliability correlation: semantics (*r* = 0.898 [0.895,0.901]), phonology (*r* = 0.709 [0.704,0.715]), and orthography (*r* = 0.700 [0.693,0.706]). In short, it appears that, although the task does not result in highly stable individual models, it gives a consistent group estimate of similarity space, with consistently superior performance for semantics across all measures.

### Determining modality uniqueness

Stimuli were presented multimodally across conditions; only the instructions differed, to focus the participant’s attention on one modality. This allowed us to test whether the multi-arrangement method can capture distinct similarity spaces for the three modalities. To verify that we were indeed describing three different and separate modalities, we compared RDMs between modalities with the same split-sample strategy used above to assess within-modality reliability. The semantic modality was found to correlate with phonology and orthography at *r* = 0.121 and *r* = 0.115, respectively. This indicates some overlap between the modalities; either semantic information was used when sorting on word form, or word form information was used when sorting by semantics, or both. Nevertheless, the result falls well short of the semantic condition’s split-half reliability to itself (*r* = 0.896), so we can say with certainty that the semantic modality was treated distinctly from the word form modalities.

Between the RDMs for phonology and orthography, however, a correlation of *r* = 0.675 is observed. This value is only slightly, though significantly, below those for each condition’s split-half reliability as noted above. This implies that participants sorted on partially unique underlying information in each condition. Although the relatively shallow Dutch spelling system would have led us to expect a high correlation between these two spaces, the participants seem to have made little distinction between phonological dissimilarity and orthographic dissimilarity.

We further evaluated if the RDMs operationalize the correct modalities by comparing them to proven computational models: Word2Vec for semantics, and Levenshtein distance of letters and phonemes for orthography and phonology, respectively. Correlating the semantic group-level RDM to dissimilarity-information taken from Word2Vec gave a correlation *r* = 0.619, whereas there were negligible correlations of the word form conditions to Word2Vec (*r* = 0.04, *r* = 0.07) and the semantic condition and Levenshtein-based models (*r* = 0.03, *r* = 0.02).

On the other hand, both form-based conditions numerically correlated more strongly with a phoneme-based model (*r* = 0.686 for phonology, *r* = 0.685 for orthography) than they did with a letter-based model (*r* = 0.628 for phonology, *r* = 0.643 for orthography). Although these values seem similarly high, it is worth mentioning that, for the stimuli used, the phoneme-based model inherently correlates with the letter-based model with a correlation of *r* = 0.818, so that any RDM correlating with one will inherently correlate strongly with the other. In sum, these varying correlational model patterns for semantics and word forms confirm that multi-arrangement can successfully capture the distinct similarity spaces.

### Understanding modality differences

**FIGURE 6:**
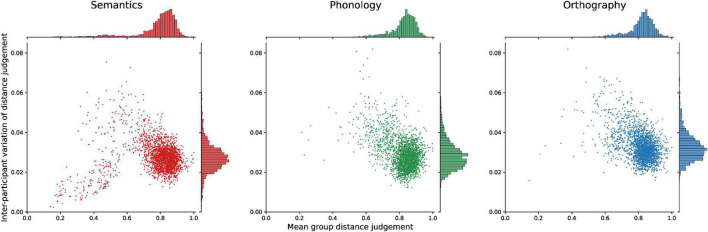
Variance in similarity judgments between participants for word pairs as a function of the word pairs’ average group dissimilarity. On the y-axis is the participants’ agreement on a word pair, with 0 indicating perfect agreement and 1 total disagreement. On the x-axis, the average dissimilarity judgment is given for each pair at the group level, with 0 indicating perfect similarity, and 1 total dissimilarity.

Next, we examined the nature of the similarity spaces that the RDMs describe (Figure 6). As seen in analyses above, the semantic condition clearly stands out from the two form-based conditions in consistency both within and between participants. A key question is whether the similarity measure is uniformly less reliable for phonology and orthography, or whether there are structural differences. To find out, we analyzed the variance in judgments over individual pairs in comparison to the average group level distance.

Across all three modalities, the group average distances of the vast majority of item pairs is distributed at the high end of dissimilarity for semantics (*M* = 0.78, SD = 0.14), phonology (*M* = 0.82, SD = 0.08), and orthography (*M* = 0.81, SD = 0.08). Participant agreement on these distances as measured by variance shows a slight trend for more agreement at high distances and less agreement (higher variance) at medium distances. Here, however, a clear difference can be seen in the semantic modality. For low dissimilarities (< 0.5; lower left corner of the plot), it shows relatively many word pairs that participants consistently judge to be very similar. This is not the case in the form-based modalities. The semantic RDM includes 167 pairs under 0.5, compared to 12 for the phonological and 20 for the orthographic RDMs. Even more distinct is the difference in agreement for such close pairs, where there is very high inter-rater agreement for items which are generally considered similar. Highly similar word pairs are clearly better captured, or more universal, for semantics than they are for phonology and orthography.

**FIGURE 7:**
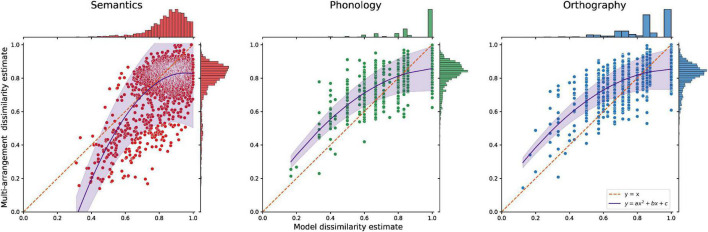
The dissimilarity of all item pairs according to each modality’s model compared to behavioral group-level multi-arrangement results. The diagonal and a simple curve fit are added to show the skew of each modality. If the multi-arrangement data and the models agreed perfectly the data should lie on the diagonal; however, multi-arrangement tends toward lower dissimilarity compared to Word2Vec, but results in higher dissimilarity compared to Levenshtein models.

To verify that these effects are not a consequence of stimulus selection or inherent differences between the modalities according to our selected models, we related the behavioral results for each item pair to the model predictions (Figure 7). According to the models underlying item selection, all three modalities should contain a range of pairs from low to high dissimilarity (see distribution plots on top). However, for semantics, the multi-arrangement results display higher similarity between pairs than was predicted by the Word2Vec model, as indicated by the bulk of items below the diagonal. This pattern is reversed for the form-based conditions, where multi-arrangement resulted in overall lower similarity between pairs than predicted by our Levenshtein models.

In sum, multi-arrangement using the IMDS algorithm can capture high similarity better for semantics than for word form. Overall, multi-arrangement seems to result in higher similarity compared to model predictions, while for word form the reported similarity is structurally lower than model predictions.

## Discussion

The goal of this study was to assess whether the multi-arrangement task can be applied to the mapping of similarity spaces for word semantics and word form (phonology and orthography). This turned out to indeed be the case, with a few qualifications. First, semantics was found to be consistently better captured than word form, especially for high similarities between items. Second, phonological and orthographic similarity spaces were not distinguishable. Third, we observed high individual variability in a similarity space largely shared at the group level. We will now discuss these findings in more detail and then evaluate the multi-arrangement method for use with form-based modalities.

### Semantic similarity captured better than form similarity

Semantic similarity was consistently better captured in the multi-arrangement task than word form similarity. This resonates with task difficulties that participants reported for the form-based conditions. The task effectively asks participants to mentally perform multidimensional scaling to create 2-dimensional arrangements. This appears to be very demanding for word form similarity even when assessing pairwise similarity would be easy. In contrast, semantic arrangements were more easily created.

One explanation is that participants have different experiences with judging similarity in the three spaces. In the semantic modality, items apparently fit in natural categories: Participants could relatively easily choose tangible dimensions on which to sort the stimuli, such as size and animacy. Participants also managed to create meaningful relationships between many items. For example, they could place “squirrel” close to other animals and “hazelnut” close to food items, while also placing “hazelnut” closer to “squirrel” than to other animals that are less likely to consume them. For the form based-modalities participants also managed to group items, for instance closely putting items together that rhyme or start with the same sequence of letters; however, grouping was less consistent between and within subjects than for semantics. Moreover, relating each item to all other items on screen seemed a more difficult task for orthographic and phonological word forms. Semantically comparing items is relatively common in daily life, both explicitly and implicitly, because it is based on taxonomies commonly agreed upon. In contrast, judging item similarities in terms of spelling is rarely done, let alone over multiple items. In sum, semantic grouping might feel more natural to participants.

Aside from the reported task difficulty, a distinct structural difference arose between sorting on semantics and on form-based modalities: For semantics, there was high agreement on highly similar items, while for word form there was little agreement on what is highly similar. This could be caused by these differences in task difficulty or lack of consensus. For example, given that there is no clear consensus on what constitutes similarity in word form, participants are free to choose and focus on particular parts of the words, resulting in different groups. Alternatively, there might be a structural difference in underlying representations: Interconnections in semantic networks could be based more on similarity than word form networks, as semantic similarity relations are critical for understanding the world, not only for selection but also for semantic generalization. Given the practical use of being able to generalize from one concept to another, or relate concepts to similar ones, judging semantic concepts on similarity might be something deeply ingrained in semantic processing. In contrast, word form interactions might be primarily competitive, to help the selection of the right word, and not wired to also generalize.

Semantic processing might be less unidirectional than word form processing, where sub-lexical units activate competing words, but once the word has been found there is no strong direct connection between competitors. Though priming effects exhibit clear similarity effects, these might be due to reactivation of primed early lexical or phonological representations, rather than direct connections between similar words. This would make it more difficult for participants to explicitly sort by the similarity we expect to see from priming studies.

Another explanation for the less consistent similarity measures for phonology and orthography, especially for expectedly similar items, could be a higher threshold for words being considered similar in the word form modalities, i.e., a higher exponential decay in the underlying similarity function as described by Shepard (1987). This could be caused by the high fidelity of orthographic representations and similarity (i.e., letters as clearly distinct units), leading to sharp representations with only very similar words being commonly perceived as similar and random idiosyncratic similarity judgments driving the other similarity judgments. Word form representations being densely clustered might be advantageous for word learning, introducing different functional constraints than for semantic representations (Dautriche et al., 2017). Semantic representations by contrast are more fuzzy: Unlike words which either contain a letter or not, semantic features like size or animacy are gradual, leading to a wider range of concepts to be generally regarded as similar, and thus to more consistent values across the similarity range (Nguyen et al., 2021).

Finally, an intriguing option at the crossroad of task demands and underlying representations is a difference in relationships between space and similarity structure for meaning and word form. Closely related semantic stimuli might be perceived as more similar, but closely related perceptual stimuli as more dissimilar (Casasanto, 2008).

To what extent differences in word form and word meaning spaces can be accounted for by any of these explanations, however, is difficult to decide in the multi-arrangement task, because here task strategies and item representations are hard to disentangle. Closer sampling across the entire spectrum of similarity, including very similar items, to map the entire similarity function, and measuring the underlying representations using neuroimaging techniques, might help discern and test the proposed explanations.

### Distinction between phonology and orthography

In our study, we observed relatively different RDMs for the semantic and form-based conditions, whereas orthographic and phonological spaces were very similar. In the latter case, both conditions correlated somewhat higher with phoneme-based Levenshtein distances than with letter-based distances. This finding by itself may suggest that participants performed the orthographic sorting task on the basis of phonology rather than orthography itself. However, we found the resulting RDMs to be slightly different, suggesting that participants did apply different criteria to each condition. These criteria, however, do not seem to be pure phoneme edit distance or letter edit distance.

The prominence of phoneme distance in sorting, even for orthographic word form similarity, might be due to the presence of diphthongs in the stimulus words that participants treated as separate units, such as ‘ui’ in Dutch. In addition, neither of our Levenshtein models assigned weights to substitutions, but participants might consider some sub-lexical items to be more similar than others (Gilmore et al., 1979). Alternative orthographic models that take these factors into account might better match participants’ subjective similarity judgments.

Similarly, our model of phoneme edit distance is the simplest that could be applied, but more sophisticated models might better capture the representational similarity of items as participants sorted them. As with orthography, there is evidence to suggest that word onset and coda disproportionately affect similarity (Hahn and Bailey, 2005), as well as evidence for not all substitutions or insertions being weighted equally (Bailey and Hahn, 2005).

### Reliable group level similarity, high individual differences

Across all modalities, similar trends arose between group-level and individual intra-rater and inter-rater agreement. The multi-arrangement task was found to provide low reliability between participants (i.e., inter-rater reliability), medium reliability within participants (i.e., intra-rater reliability), but high consistency at the group level (i.e., split-half reliability) in all three modalities. This finding is in line with previous semantic studies (e.g., Richie et al., 2020). One initial interpretation is that the data are rather noisy at the individual level and do not provide proper estimates based on the limited number of trials available.

Alternatively, and more in line with the higher intra- than inter-rater reliability, major individual differences may exist in the underlying psychological space of the participants, which nevertheless are all weakly related to a shared universal space.

Intra-rater reliability measures provide some insight into the role of individual differences, as they are higher than inter-rater reliability measures. In all, the group average RDMs appear to represent some universally agreed similarity information, but cannot account for a significant portion of each individual’s unique judgments. Observed individual differences may be a consequence of both underlying representational differences and differing task strategies.

As attempts to cluster participants statistically or though visual inspection of their employed strategies have failed, we conclude that different approaches to the task do not seem to result in different similarity outcomes. Although we could not statistically discern participant groups with different task strategies, participants might nonetheless begin to associate items on different dimensions early in the session. Their specific initial choices might give strong weight to certain relationships between items, even though their underlying representations may be similar to those of other participants. Differences in task strategy also do not appear to significantly affect intra-participant reliability.

The finding of high split-half reliability proves that group level RDMs provide stable representations. For the semantic condition, split-half reliability was higher than the correlation to the Word2Vec. As such, the multi-arrangement task accounts for some similarity information that the corpus-based Word2Vec model does not.

In sum, the multi-arrangement task generated useful group-level data for our multimodal stimulus set across all three item dimensions, but showed high individual differences. The role of individual differences in underlying similarity representations is difficult to disentangle from differences in task strategy and noise. Fewer stimuli or more time per participant might be needed to arrive at stable individual RDMs, while modeling efforts could help make concrete predictions about the three sources of variation.

### Limitations and further directions

In addition to the recommendations mentioned before, we see a number of limitations and directions for further study. A general concern, even for unimodal semantic application, is the assumption that large distances on screen are most informative. This assumption leads to the desirable zoom-in effect, which lets participants further specify item relationships within clusters. However, we also noticed that participants are more careful about the relative placement of items within clusters than between clusters. That is, highly similar words were sorted close together with more care for relative distances, than the place of that group compared to other groups. Consistently, the relatively high agreement on similarity for highly similar item pairs also suggests that small on-screen distances might be the most informative, with more noisy information being contained in large screen distances.

Unfortunately, we cannot analyze whether a different metric would be more suited, as the algorithm is applied during the experiment to select the next trial stimulus set on the fly and is based on currently completed trials. The algorithm is therefore a baked-in co-determiner of the resulting data. Further investigation could assess if a different similarity metric can produce more informative trials or lead to better trial weighting for the final RDM construction. To evaluate this issue, data be collected with fully randomized trials, accepting a loss of the efficiency gained through on-the-fly evidence estimation.

The choice to present multimodal stimuli in matched sessions that only differed in task instructions was validated by the finding that participants sorted based on unique criteria across conditions. The negligible correlation between semantic and form based RDMs confirms that there was little influence of one modality on the sorting of another, despite all being presented at once. While this could have occurred in case of unclear task instruction, it is also important to note that this means participants were not subconsciously affected by, for example, semantic similarities when trying to sort by phonology. The low observed correlation between semantic and form-based RDMs might be the result of negligible task confusion, but could also be representing true correlations between the underlying representations where form-meaning consistency exists (Marelli et al., 2015; Cassani and Limacher, 2022). This is crucial to enable extension of the task to the phonological modality, as spatial sorting of solely auditory stimuli is not possible. Participants need a visual cue for each item they sort, and apparently can avoid confounding their sorting by semantic similarity when instructed to do so.

Naturally our stimulus selection is limited by investigating all three modalities in one experiment. Monomodal applications of the multi-arrangement tasks could use more a naturalistic word selection including more varying word lengths, word forms other than nouns, and more abstract (less imageable) words. The effect of polysemous or more ambiguous words is also left open by this study. It may be possible to account for polysemy by introducing a preliminary task in which participants roughly categorize each item (Majewska et al., 2020).

As discussed, the data revealed large individual differences in perceived similarities. We cannot definitively conclude whether this finding represents idiosyncratic task performance or idiosyncratic underlying representations, because we did not collect similarity information from the same participants by alternative methods. For representational similarity analysis, this implies that behavioral sorting data may need to be collected from the same participant as the neuroimaging data to which it is to be compared to take into account these idiosyncrasies. In fact, a combination of group level RDM and personal RDM might result in the least-noisy, most individual similarity space to be used for comparison to any one participant.

Our evidence is in line with current research on lexical representation and processing and shows that the multi-arrangement method can be used to measure representations across multiple modalities. We can conclude that semantic and form representations are largely orthogonal, with some overlap either due to coactivation or due to limited form-meaning correlation in the underlying representations. Although we know that representations across all modalities are coactivated in language use, subjects were able to inhibit other modalities while sorting.

## Conclusion

The multi-arrangement method for collecting similarity data was successfully used to collect similarity data on semantics and word form. Importantly, the method generated stable group-level similarity models, yet revealed large idiosyncratic differences in similarity judgments for all modalities. Over all measures, semantic similarity estimates were more consistent than measures of word form similarity, especially for highly similar items. This suggests potential differences between modalities in the applicability of the method, subject strategy and/or underlying representations, important to keep in mind when applying the multi-arrangement method and studying multimodal lexical representations.

## Glossary

IMDS, Inverse Multidimensional Scaling, a process by which multiple trials from one participant are aggregated into a single RDM.

RDM, Representational Dissimilarity Matrix, a matrix containing the dissimilarity estimate between any pair of stimuli.

## Data availability statement

The datasets presented in this study can be found in online repositories. The names of the repository/repositories and accession number(s) can be found below: https://doi.org/10.34973/6ats-e232.

## Ethics statement

The studies involving human participants were reviewed and approved by Radboud University Social Sciences Ethics Committee. The patients/participants provided their written informed consent to participate in this study.

## Author contributions

LA performed the programming, data collection, analysis, and figures with feedback from the other authors. All authors contributed to the concept and design of the study, contributed to the article, and approved of the submission.
