# Supplementary material for: Characterizing the semantic and form-based similarity spaces of the mental lexicon by means of the multi-arrangement method
Source: Front Psychol. 2022 Aug 11;13:945094. doi: 10.3389/fpsyg.2022.945094 (PMC9407019; doi:10.3389/fpsyg.2022.945094)
Supplement: Supplementary file 1 [file Data_Sheet_1.PDF]

Supplementary Material

1 Stimuli

The experiment requires 70 Dutch words in written, spoken, and image form. Only object nouns were used. To assure that consistent and tested images are available, words were selected from the international picture naming project (Székely et al., 2003).

In order for words to have roughly the same written and spoken length, they were restricted to words with 4 to 8 letters and exactly 2 syllables. Including longer words would introduce practical issues with presenting stimuli uniformly in the sorting task, as well as potentially introducing a confound of word length on task difficulty and potentially additional variance between subjects on how to treat differences in word length. A few additional words were excluded from the selection for having an ambiguous Dutch translation, i.e. where naming the picture in Dutch was not expected to be consistent. Polysemous Dutch words were also excluded as it would require participants to arbitrarily choose one meaning to sort on or might lead participants to switch which meaning they sort on between trials.

Of the remaining 165 valid words from the IPNP, 7 word groups of 10 words each were systematically selected to maximize variance in orthographic, phonological, and semantic distance between words in each group. Orthographic and phonological distances were measured as normalized Levenshtein distance. Phonemes were acquired in the PhonCLX format from the CELEX database (Max Planck Institute for Psycholinguistics, 2001). Semantic distances were taken from the Google News Corpus using Word2Vec (Mikolov et al., 2013) for English translation equivalents of the Dutch stimuli. The procedure can be found under experiment/preprocessing/wordSelection/wordSelection.py in the project repository.

All groups were tested through one-way ANOVA on their word frequencies, log10 frequencies, and word length to avoid confounding factors, using data from SUBTLEX (Keuleers et al., 2010). Due to correlation between semantic distance in Word2Vec and word frequency, word groups do have noticeably different average frequencies. They were not, however, statistically significant.

1.1 Audio

Audio was recorded in the Music lab of the BSI at Radboud University. A female native Dutch speaker read the words. Recordings were adjusted for volume and pitch, and each word was recorded three times in random order to select the most clearly pronounced iteration.

1.2 Images

The images can be found on the repository under experiment/stimuli/. Most are from the IPNP, although some had to be replaced for copyright reasons. All images are inverted to white lines on black background.

2 Words

Group 1	Group 2	Group 3	Group 4	Group 5	Group 6	Group 7
radijs	schommel	handdoek	eenhoorn	ober	ballon	pijler
appel	kalkoen	ladder	spiegel	koffer	vlinder	trechter
eikel	trompet	douche	vleugel	mand	kikker	lama
koning	beha	piraat	vlieger	pinguïn	vinger	pijl
hengel	varken	viool	zwembad	wortel	fluitje	kano
perzik	konijn	hanger	tijger	pinda	ridder	banjo
aardbei	walvis	anker	fornuis	radio	balkon	banaan
stekker	kanon	luier	eekhoorn	panda	pincet	citroen
geweer	trommel	zeehond	sleutel	dokter	mixer	hangmat
tomaat	walrus	masker	vogel	boter	potlood	gieter

Table 1. The 70 selected words used for stimuli in this study.

3 Additional statistics

Section	Measure	Pearson	Spearman
4.2 Consistency of similarity spaces	Inter-rater agreement: Semantics	0.235	0.187
	Inter-rater agreement: Phonology	0.080	0.061
	Inter-rater agreement: Orthography	0.077	0.060
	Intra-rater agreement: Semantics	0.530	0.486
	Intra-rater agreement: Phonology	0.332	0.288
	Intra-rater agreement: Orthography	0.309	0.268
	Individual vs group: Semantics	0.471	0.329
	Individual vs group: Phonology	0.258	0.187
	Individual vs group: Orthography	0.252	0.182
	Split-half reliability: Semantics	0.898	0.663
	Split-half reliability: Phonology	0.709	0.528
	Split-half reliability: Orthography	0.700	0.510
4.3 Determining modality uniqueness	Semantics vs Phonology	0.121	0.166
	Semantics vs Orthography	0.115	0.156
	Phonology vs Orthography	0.675	0.611
	Semantics vs Word2Vec	0.619	0.313
	Phonology vs Word2Vec	0.041	0.034
	Orthography vs Word2Vec	0.069	0.073
	Phonology vs Phoneme Levenshtein	0.686	0.530
	Orthography vs Phoneme Levenshtein	0.685	0.541

Table 2. Pearson and Spearman correlation comparisons.

4 Additional Analyses

We used English translation equivalents of the Dutch words to collect semantic vectors from the Word2Vec model mentioned above. Here we also compare the collected data against a Dutch corpus trained Word2Vec model (Nieuwenhuijse, 2018), as well as comparing the two models against each other.

Measure	Pearson	Spearman
Semantics vs English Word2Vec *	0.619	0.313
Semantics vs Dutch Word2Vec	0.602	0.337
English Word2Vec vs Dutch Word2Vec	0.691	0.571

Table 3. Comparison to a Dutch Word2Vec model.

We additionally used similarity data based on feature norms collected by Buchanan (2018):

Measure	Pearson	Spearman
Semantics vs Word2Vec *	0.619	0.313
Semantics vs Word Pair Norms	0.425	0.321
Word2Vec vs Word Pair Norms	0.442	0.351

Table 4. Comparison against the Buchanan feature norm dataset.

Images were used as a proxy to lexical semantics. We explicitly instructed participants to use the image as a stand-in for the semantic concept, and not sort on visual similarity of the line drawings. To verify how much of the visual similarity between images was taken into account by the participants, we applied the VGG-16 classifier, similar to the VGG-F model used by Günther et al (2020), to our data, and trained it on the same ImageNet stimulus set, to measure visual similarity between our line drawings.

Measure	Pearson	Spearman
Semantics vs VGG16	0.161	0.053

Table 5. Comparison with VGG16 model.

We also compared the orthographic and phonological RDMs against the weighted open bigram model (Whitney & Cornelissen, 2008) and Fischer-Baum's extension of Davis' spatial coding model (Davis, 2010; Fischer-Baum et al., 2011) based on letters or phonemes respectively. Both models were implemented in Python by Rick Dijkstra. The findings show a similar pattern, with seemingly

superior performance for Open Bigrams, inviting further research to delineate the optimal way to capture human orthographic similarity judgments.

Measure	Pearson	Spearman
Orthography vs Levenshtein *	0.643	0.521
Orthography vs Open Bigrams	0.723	0.523
Orthography vs Spatial Coding	0.678	0.497
Phonology vs Phoneme Levenshtein *	0.686	0.530
Phonology vs Phoneme Open Bigrams	0.714	0.504
Phonology vs Phoneme Spatial Coding	0.689	0.528

Table 6. Alternative word form models.

To investigate whether agreement between our group-level data and models differs for only close pairs, we ran the reported analyses with only the subset of item pairs with a dissimilarity of 0.6 or lower according to each model. This produces nominally slightly different results for the word form modalities, and an interesting flip between Pearson and Spearman correlations for semantics, indicating that for similar items, Word2Vec and the behavioral data agree more monotonically and less linearly.

Measure	Subset	Pearson	Spearman
Semantics vs Word2Vec *	2415	0.619	0.313
Semantics vs Word2Vec Close Pair Subset	98	0.490	0.545
Phonology vs Phoneme Levenshtein *	2415	0.686	0.530
Phonology vs Phoneme Levenshtein Close Pair Subset	90	0.649	0.512
Orthography vs Levenshtein *	2415	0.643	0.521
Orthography vs Levenshtein Close Pair Subset	179	0.593	0.436

Table 7. Comparison of high-similarity pair subsets.

* The primary analysis used in results for this paper.

5 References

Buchanan, E. M. (2018). *Word Norms Paired Word Information* [Data set]. https://zenodo.org/badge/latestdoi/116844736

Davis, C. J. (2010). The spatial coding model of visual word identification. *Psychological Review*, *117*(3), 713–758. https://doi.org/10.1037/a0019738

Fischer-Baum, S., Charny, J., & McCloskey, M. (2011). Both-edges representation of letter position in reading. *Psychonomic Bulletin & Review*, *18*(6), 1083–1089. https://doi.org/10.3758/s13423-011-0160-3

Günther, F., Petilli, M. A., Vergallito, A., & Marelli, M. (2020). Images of the unseen: extrapolating visual representations for abstract and concrete words in a data-driven computational model. *Psychological Research*. https://doi.org/10.1007/s00426-020-01429-7

Keuleers, E., Brysbaert, M., & New, B. (2010). SUBTLEX-NL: a new measure for Dutch word frequency based on film subtitles. *Behavior Research Methods*, *42*(3), 643–650. https://doi.org/10.3758/BRM.42.3.643

Max Planck Institute for Psycholinguistics. (2001). *CELEX Database*. WebCelex. http://celex.mpi.nl/

Mikolov, T., Chen, K., Corrado, G., & Dean, J. (2013). Efficient Estimation of Word Representations in Vector Space. In *arXiv [cs.CL]*. arXiv. http://arxiv.org/abs/1301.3781

Nieuwenhuijse, A. (2018). *Coosto - Dutch Word Embeddings* (Version 1.2.0) [Computer software]. Coosto. https://github.com/coosto/dutch-word-embeddings

Székely, A., D’Amico, S., Devescovi, A., Federmeier, K., Herron, D., Iyer, G., Jacobsen, T., & Bates, E. (2003). Timed picture naming: extended norms and validation against previous studies. *Behavior Research Methods, Instruments, & Computers: A Journal of the Psychonomic Society, Inc*, *35*(4), 621–633. https://www.ncbi.nlm.nih.gov/pubmed/14748507

Whitney, C., & Cornelissen, P. (2008). SERIOL Reading. *Language and Cognitive Processes, 23*(1), 143–164. https://doi.org/10.1080/01690960701579771
